# Supercritical fluid in deep subduction zones as revealed by multiphase fluid inclusions in an ultrahigh-pressure metamorphic vein

**DOI:** 10.1073/pnas.2219083120

**Published:** 2023-05-08

**Authors:** Deshi Jin, Yilin Xiao, Dong-Bo Tan, Yang-Yang Wang, Xiaoxia Wang, Wancai Li, Wen Su, Xiaoguang Li

**Affiliations:** ^a^Key Laboratory of Crust-Mantle Materials and Environments of the Chinese Academy of Sciences, School of Earth and Space Sciences, University of Science and Technology of China, Hefei 230026, China; ^b^Center of Excellence in Comparative Planetology of the Chinese Academy of Sciences, Hefei 230026, China; ^c^Key Laboratory of Lithospheric Evolution, Institute of Geology and Geophysics, Chinese Academy of Sciences, Beijing 100029, China

**Keywords:** multiphase fluid inclusions, supercritical fluid, UHP metamorphic vein, subduction zones

## Abstract

Due to their low viscosity, high mobility, and high element contents, supercritical fluids are important agents in the cycling of elements. However, the chemical composition of supercritical fluids in natural rocks is poorly understood. Here, we investigate well-preserved primary multiphase fluid inclusions (MFIs) from an ultrahigh-pressure (UHP) metamorphic vein of the Bixiling eclogite in Dabieshan, China, thus providing direct evidence for the components of supercritical fluid occurring in a natural system. Via the 3D modeling of MFIs by Raman scanning, we quantitatively determined the major composition of the fluid trapped in the MFIs. Combined with the peak-metamorphic pressure–temperature conditions and the cooccurrence of coesite, rutile, and garnet, we suggest that the trapped fluids in the MFIs represent supercritical fluids in a deep subduction zone. The strong mobility of the supercritical fluids with respect to carbon and sulfur suggests that such fluids have profound effects on global carbon and sulfur cycling.

## Significance

Our research presents a method that more accurately determines the quantitative composition of ultrahigh-pressure (UHP) fluid [high H_2_O (~40 wt.%) and solute (~60 wt.%) contents] released by a slab during deep subduction compared with that detailed in previous studies. The data provide important information for understanding the characteristics of UHP fluids, especially supercritical fluids. Supercritical fluids with high dissolved contents of carbon (2 wt.%) and sulfur (3 wt.%) released during deep subduction could cause efficient migration of carbon and sulfur from slabs. This improves their recycling efficiency in the subduction zone, playing a vital role in the deep cycling of these elements. The contribution of supercritical fluids to the deep carbon and sulfur cycle may have been significantly underestimated previously.

Fluids released from slabs at different subduction depths may show large differences in chemical composition and speciation, and they can be subdivided into hydrous melts, aqueous fluids, and supercritical fluids, with the latter being released at high-temperature and high-pressure (HP) conditions (1). Their high mobility and low viscosity make supercritical fluids (2) effective carriers of elements from subduction zones. Therefore, the formation and evolution of supercritical fluid plays a significant role in the circulation of material in the earth, particularly for subduction-related magmatism and mineralization processes (3). However, the chemical characteristics of the fluid remain poorly understood due to a lack of suitable samples and the complexities of in situ analysis.

The immiscibility of supercritical fluid under decreasing temperature and pressure conditions (4) and the interactions with surrounding rocks decrease the likelihood that supercritical features will be preserved. Nevertheless, high pressure–temperature experimental studies have provided second critical end points (SCEP) for several rock–H_2_O systems (3, 5, 6). However, because of experimental technique limitations, a general consensus has not been reached on the SCEP of related rock–H_2_O systems (3, 5), especially basalt–H_2_O system. Nonetheless, considerable evidences have been found for supercritical fluid activity in natural rock, especially ultrahigh-pressure (UHP) inclusions. For continental subduction zones, multiphase inclusions that may be related to supercritical fluids have been documented in multiple metamorphic belts worldwide. The typical multiphase solid–fluid inclusions in Dabie-Sulu (7–10), Dora-Maria (11), and Rhodope (12) are considered as supercritical fluid or its remnants. In addition, some water-free multiphase solid inclusions (MSIs) can be regarded as the residual melt phase of supercritical fluid after it appears immiscible or reacts with surrounding rock, e.g., diamond-bearing MSIs in Erzgebirge (13) and Kokchetav (14) UHP metamorphic rocks are thought to be the products of supercritical fluids of COH–silicate and COH–sulfide–silicate systems, respectively. These inclusions are significantly different than felsic MSIs, which represent partial melt products. The multiphasefluid inclusion (MFIs) (15) in the Lago di Cignana belt also documented the activity of supercritical fluid in subducted oceanic crust. Furthermore, such water-free MSIs in peridotite that represent residual reactions are not rare (16, 17). The cited studies have laid the foundation for the identification of supercritical fluid. However, the methods lack the support of quantitative and reliable petrographic and geochemical indicators. Consequently, our understanding of supercritical fluid in subduction zones mainly relies on indirect observations and semi-quantitative estimations from related UHP veins and metasomatic peridotite (10, 17, 18). Although the supercritical state is not maintained in inclusions, the host minerals provide a relatively closed environment for supercritical fluid to retain its original chemical characteristics. MFIs are the most likely candidates of relic primitive supercritical fluid and have great potential in the identification and reconstruction of the composition of supercritical fluid.

Here, we report the MFIs in omphacite and garnet in a metamorphic vein of the Bixiling UHP eclogite from central Dabieshan, which provide an exceptional window into the quantitative composition of supercritical fluids. 3D Raman scanning and modeling allow us to clarify the chemical characteristics of the supercritical fluids and further examine the results of experimental petrology. Analytical data and modeling calculations showed that the MFIs represent a directly documented occurrence of supercritical fluid released by a deeply subducted slab presenting unique chemical characteristics. Hence, we determined that MFIs provide direct evidence of supercritical fluid released by deeply subducted slab and show that supercritical fluid may play a more important role in subduction material cycling than previously reported.

## Results and Discussion

**Fig. 1.**
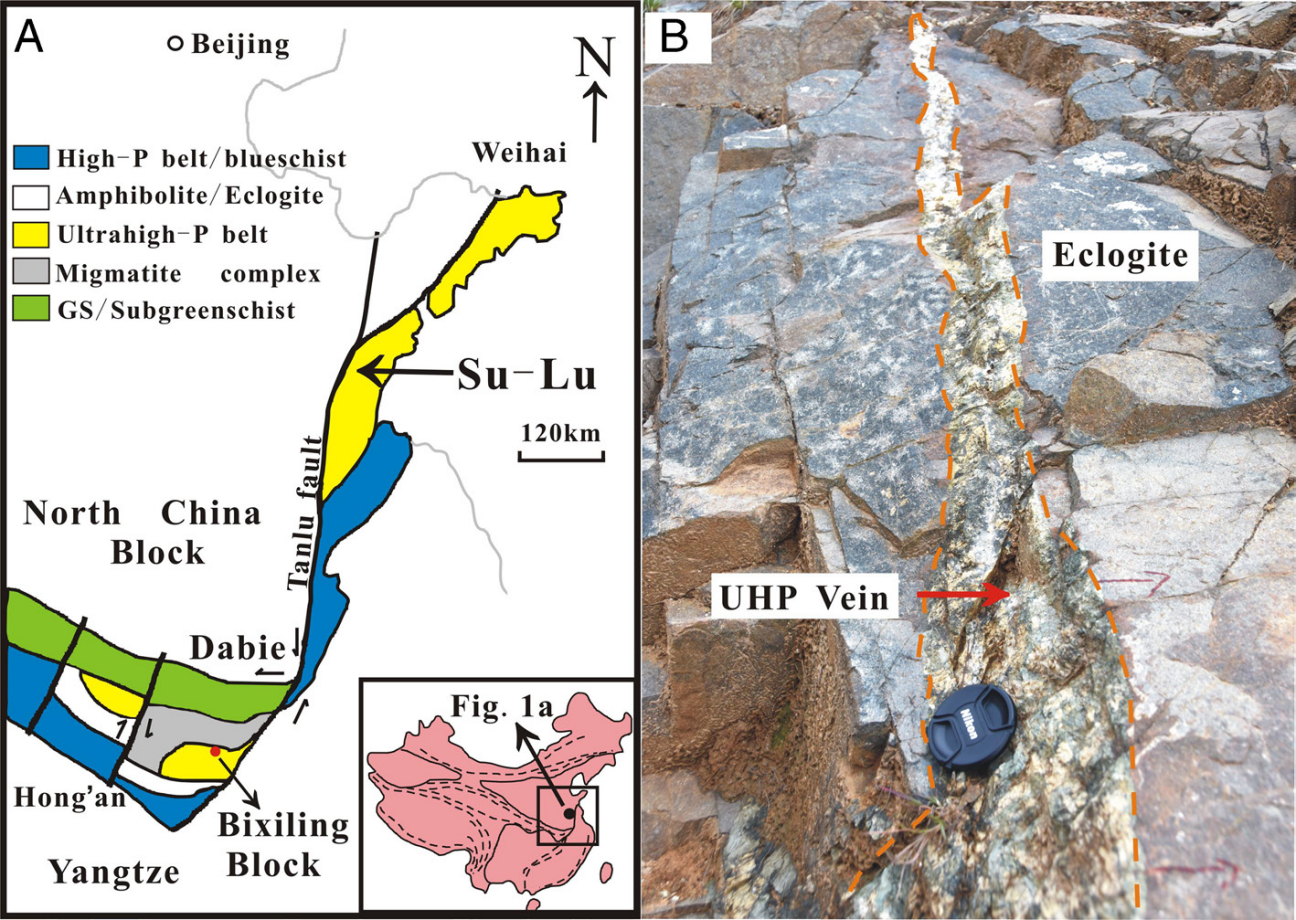
(*A*) Geological sketch map of the Dabie-Sulu orogenic belt. The maps are modified based on a study by Huang et al. (20). (*B*) Field relationship between the UHP vein and the host eclogite at the sampling point.

**Fig. 2.**
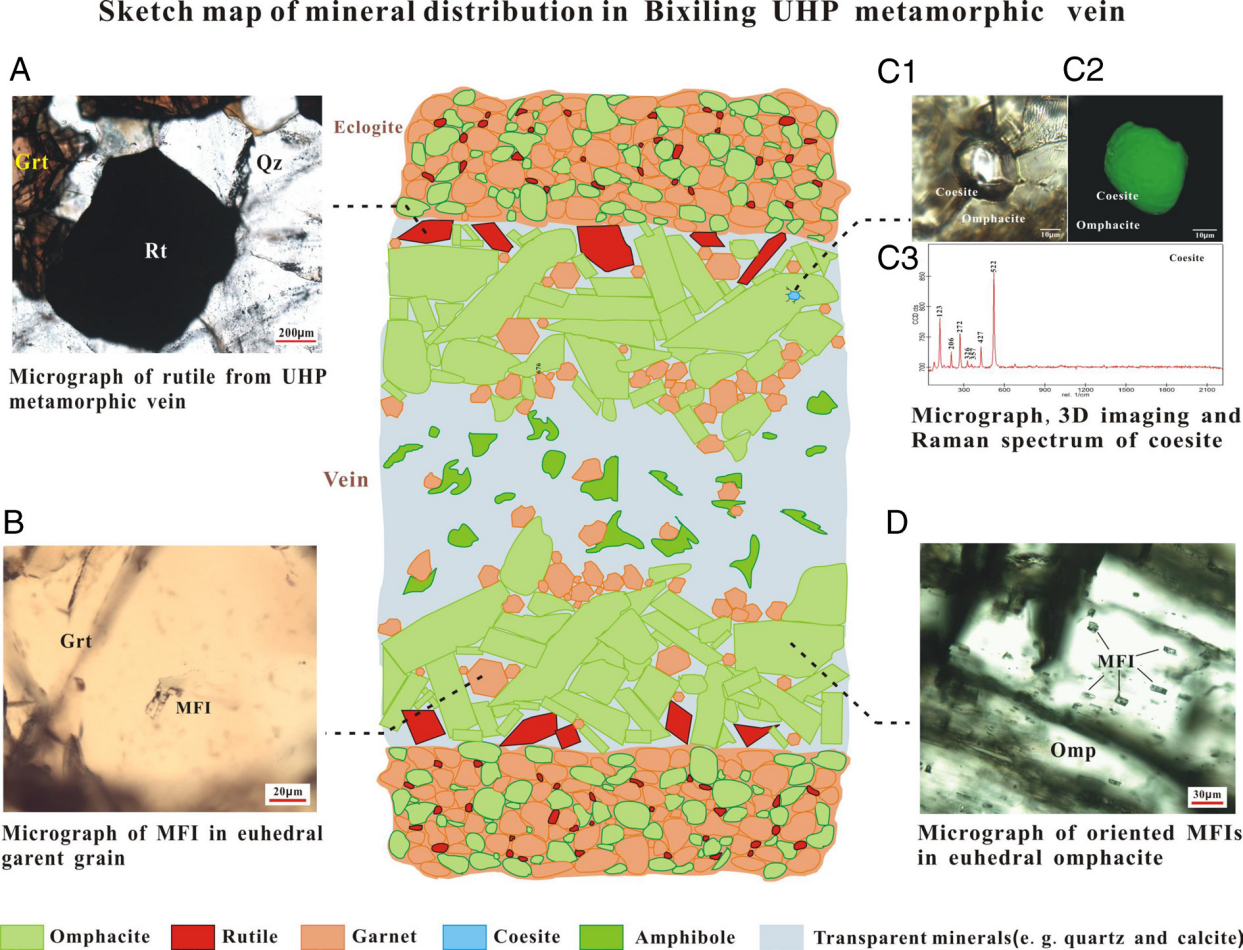
Sketch map of the mineral distribution of the Bixiling UHP metamorphic vein based on petrographic observations of multiple vein sections. (*A*) Rutiles occur as large euhedral grains along the vein wall. (*B*) Isolated MFIs within euhedral garnet. (*C*) Coesite with perfect crystal form exists as mineral inclusions in the omphacite of the UHP vein. 3D image by Raman scanning shows complete coesite grain without quartz retrograde edge. (*D*) Array of primary oriented inclusions within omphacite.

Metamorphic HP-UHP veins are generally considered a direct record of deep fluid activity in subduction zones. Previous studies of the UHP metamorphic rocks in the Dabie-Sulu area indicated that the internal source of the fluids forming HP veins was associated with dehydration (19, 20). A large number of highly saline fluid inclusions containing NaCl daughter minerals in the peak metamorphic minerals of Dabie-Sulu were considered the product of late supercritical fluid rebalancing (21–23). In this study, we chose a lenticular vein hosted in the eclogite of the Bixiling complex for investigation. Located in western Anhui Province, the Bixiling eclogite is the largest coesite-bearing mafic-ultramafic body in the Dabie-Sulu orogenic belt, which occurs as a tectonic block with an outcropping area of 1.5 km^2^ within foliated quartz-feldspathic gneiss (20). The complex consists predominantly of eclogites with a few garnet-bearing ultramafic lenses. Inclusions of coesite relicts, magnesite, and Ti-clinohumite in eclogite minerals suggest subduction depths of >100 km and peak-metamorphic conditions of up to 700 to 800 °C and >4.0 GPa (21) at an age of approximately 220 Ma (24). We collected two host eclogite samples and six vein samples rich in omphacite situated at the lower end of the vein (Fig. 1). The samples consist predominantly of omphacite, garnet, quartz, and calcite with rutile, apatite, and hornblende. The grain size of these minerals is larger and better developed compared to the morphology of the same minerals in the surrounding eclogite. These characteristics show that the vein minerals directly crystallized from the vein-forming fluid and were not removed from the host rock and transported as loose crystals in the fluid (*SI Appendix*, Fig. S1). Omphacite and rutile are mainly distributed along the vein wall in the form of large euhedral grains (1 to 3 cm) (Fig. 2). Coesite occurs as mineral inclusions in omphacite from the UHP vein (Fig. 2*C*), with radial cracks at the edge, thus confirming vein formation within the stability field of coesite, i.e., >2.8 GPa.

The vein has a sharp and distinct boundary with the surrounding eclogites, without an evident reaction halo. A previous study also supports this observation and showed that the silicate minerals and newly grown zircons in the vein show similar O isotope and Hf isotope compositions to those in the eclogites (25). These results indicate that the fluid responsible for the UHP veining is internally buffered within the UHP eclogites (25)**.** The vein-forming fluid should be in equilibrium with the host eclogites and not present reactivity. Therefore, the studied metamorphic vein represents a relatively primitive UHP fluid released during deep subduction.

**Fig. 3.**
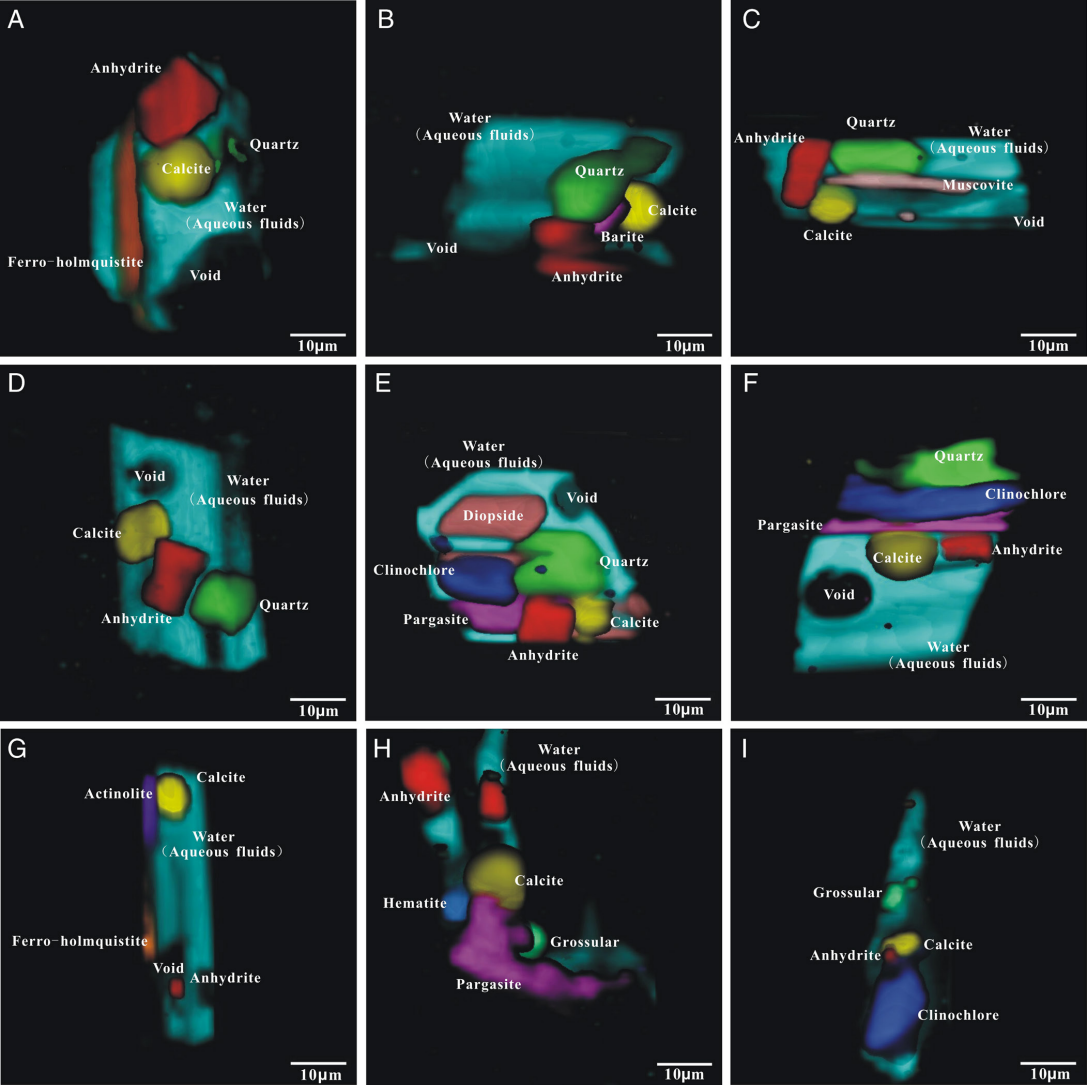
3D images of the MFIs (for photomicrographs, see *SI Appendix*, Fig. S4) from the UHP complex metamorphic vein. (*A*–*G*) MFIs within omphacite. (*H* and *I*) MFIs showing the characteristics of fluid leakage in garnet.

A large number of MFIs are found in omphacite, and they show a preferred directional distribution that is parallel to mineral elongation (Fig. 2*D*). Most inclusions are 10 to 40 μm in diameter and show nearly perfect negative crystal shape without obvious signs of postentrapment modifications (Fig. 3). MFIs with cracking in omphacite also exist and occur in symbiosis with noncracking inclusions. The decrepitation of MFIs only led to fluid leakage without significant changes in the assemblage of daughter minerals (*SI Appendix*, Fig. S6). Moreover, MFIs in garnet are far fewer and more irregular than those in omphacite. These healed cracks filled with H_2_O and signs of fluid leakage (e.g., surrounding tiny fluid inclusions) (*SI Appendix*, Fig. S4 *H* and *I*) indicate that the primary inclusions were cracked during postentrapment. We believe a possible mechanism is that unlike omphacite, which only forms under metamorphic conditions above eclogite-facies, garnet grows over a wide range of temperatures (as low to 400 °C) and pressure (amphibolite-facies). This means that garnet in metamorphic veins could have experienced complex multiple growth stages. Therefore, the UHP MFIs trapped in early-stage garnet were most likely to be affected by the later growth (extrusion deformation) of the mineral, which resulted in the very common occurrence of postentrapment modifications or fluid leakage of UHP MFIs in garnet.

The Raman analysis results (*SI Appendix*, Fig. S2) demonstrate that the daughter mineral assemblages are similar for all suitable fluid inclusions (~40), including quartz, calcite, sulfates (anhydrite, barite), silicates (e.g., muscovite, clinochlore, pargasite), and H_2_O (*SI Appendix*, Fig. S3) in omphacite and anhydrite, calcite, silicates (grossular, clinochlore, pargasite), hematite, and H_2_O in garnet. The newly developed 3D Raman scanning and modeling allowed us to clarify the chemical characteristics of supercritical fluid and further examine data obtained from laboratory experiments. Nine inclusions with good morphology and large size were selected for 3D imaging and modeling analysis. 3D imaging (Fig. 3) demonstrated the distribution and relationship of each daughter mineral phase. Each daughter mineral in the MFIs with total fill degrees >95 % was mostly idiomorphic and had a similar phase proportion. In addition to abundant water (H_2_O), occasional small cavities could be observed within the inclusions, and they were likely caused by volume contraction during cooling.

The results of 3D modeling and calculations (detailed process and raw data can be found in *Methods* and *SI Appendix*, Table S1) indicate constant bulk chemical compositions of the inclusions in omphacite (Table 1). The SiO_2_ contents range from 12.9 wt.% to 31.5 wt.%, but typically fall around 25 wt.%, and the content of H_2_O is always around 45 wt.%. The other components showed less variations, with the CaO content ranging from 11.1 to 17.8 wt.%, SO_3_ content ranging from 2.7 to 14.6 wt.%, and CO_2_ content ranging from 3.6 to 8.3 wt.%. Minor components of MgO, Al_2_O_3_, and TFe_2_O_3_ had values less than 6.0 wt.%, while those of Li, Na, and K had values of approximately 0.1 wt.%. Compared to omphacite, the MFIs in garnet had lower contents of SiO_2_ (13.4 to 19.1 wt.%) and higher contents of MgO (5.3 to 18.7 wt.%), Al_2_O_3_ (5.3 to 11.8 wt.%), and TFe_2_O_3_ (9.3 to 11.0 wt.%). The MFIs in the omphacite and garnet had the same general H_2_O content.

**Table 1.** Summary table of calculated compositions of trapped fluids of the MFIs

Host	MFI	Mineral assemblages (wt.%)	CaO (%)	BaO (%)	SiO_2_ (%)	SO_3_ (%)	CO_2_ (%)	Na_2_O (%)	Al_2_O_3_ (%)	MgO (%)	TFe_2_O_3_ (%)	Li_2_O (%)	K_2_O (%)	H_2_O (%)
Omphacite	1	Cc(8.2%)+Qz(21.7%)+Ahy(17.4%) +Fe_2_-Hq(8.9%)+H_2_O(43.8%)	11.7	–	26.8	10.2	3.6	–	1.1	–	2.3	0.2	–	44.1
	2	Cc(9.4%)+Qz(21.4%)+Ahy(15.4%) +Brt(9.0%)+H_2_O(44.8%)	11.1	5.7	24.5	11.7	4.0	–	–	–	–	–	–	43.0
	3	Cc(12.4%)+Qz(27.3%)+Ahy(15.2%) +Ms(9.1%)+H_2_O(36.0%)	13.2	–	31.5	8.9	5.4	–	3.5	–	–	–	1.1	36.4
	4	Cc(13.6%)+Qz(19.2%)+Ahy(24.8%) +H_2_O(42.4%)	17.8	–	19.2	14.6	6.0	–	–	–	–	–	–	42.4
	5	Cc(7.2%)+Qz(14.2%)+Ahy(6.7%)+Prg(7.8%) +Clc(5.7%)+Di(16.0%)+H_2_O(42.4%)	12.0	–	28.3	3.9	3.2	0.3	2.5	6.5	–	–	–	43.4
	6	Cc(13.8%)+Qz(16.5%)+Ahy(4.7%)+ Prg(11.8%)+Clc(17.1%)+H_2_O(36.1%)	11.2	–	27.2	2.7	6.1	0.4	5.3	8.4	–	–	–	38.7
	7	Cc(18.9%)+Act(14.4%)+Ahy(8.0%)+ Fe_2_-Hq(9.0%)+H_2_O(49.7%)	15.7	–	12.9	4.7	8.3	–	1.1	1.6	5.2	0.2	–	50.2
	Average		13.2	0.8	24.3	8.1	5.2	0.1	1.9	2.4	1.1	0.1	0.2	42.6
Garnet	8	Cc(21.8%)+Ahy(14.0%)+Grs(5.4%)+ Prg(41.0%)+Hem(6.7%)+H_2_O(11.1%)	25.5	–	19.9	8.2	9.6	1.5	8.7	7.9	6.7	–	–	12.0
	8^*^	Cc(14.7%)+Ahy(9.4%)+Grs(3.7%)+ Prg(27.7%)+Hem(4.5%)+H_2_O(40.0%)	17.2	–	13.4	5.6	6.5	1.0	5.9	5.3	4.5	–	–	40.6
	9	Cc(10.8%)+Ahy(1.7%)+Grs(5.4%) +Clc(52.3%)+H_2_O(29.8%)	8.8	–	19.1	1.0	6.1	–	10.8	18.7	–	–	–	35.5
	Average		13.0	–	16.3	3.3	6.3	0.5	8.4	12.0	2.3	–	–	38.1
AVERAGE			13.2	0.6	22.5	7.0	5.5	0.2	3.4	4.5	1.3	0.0	0.1	41.6

^*^Corrected composition of MFIs. Inclusions 1 to 9 correspond to the inclusions of Fig. 3 (*A*–*I*.) The abbreviated mineral stands for the following: Cc: calcite; Qz: quartz; Ahy: anhydrite; Fe2-Hq: ferro-holmquistite; Prg: pargasite; Act: actinite; Brt: barite; Ms: muscovite; Clc: clinochlore; Di: diopside; Grs: grossular; Hem: hematite.

As typical eclogite-facies minerals, omphacite and garnet are the earliest minerals that crystallize from vein-forming fluid. Petrological criteria, including the negative crystal shape and directional distribution parallel to mineral elongation of the MFIs in omphacite, indicate the primary origin of the MFIs. Although the morphology of MFIs in garnet is less regular compared to the inclusions in omphacite, they can be classified as primary as well. The mineral assemblages and trapped fluid composition of MFIs in garnet and omphacite are comparable, suggesting the same source.

**Fig. 4.**
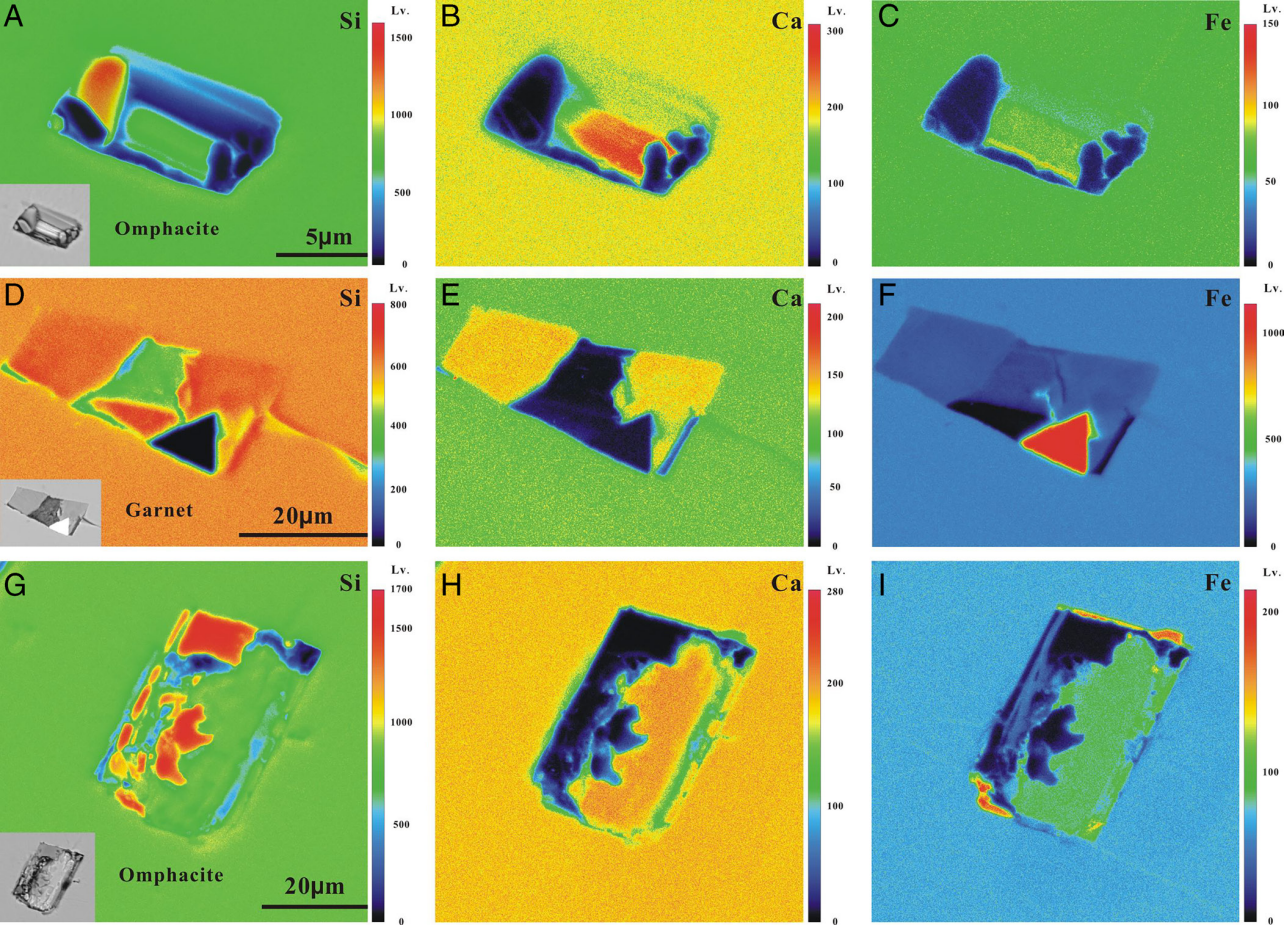
Major element mapping of Si, Ca, and Fe of MFIs in omphacite and garnet. Three MFIs were selected for destructive polishing and Electron Probe mapping analysis, and their size, morphology, and daughter mineral assemblages are consistent with those of modeled MFIs (Fig. 3). The images in the lower left of (*A*, *D*, and *G*) are BSE photographs of the three analyzed MFIs. (*A*–*C* and *G*–*I*) Major element mappings of Si, Ca, and Fe in two MFIs in omphacite. (*D*–*F*) Major element mappings of Si, Ca, and Fe of the MFIs in garnet. The crack in the inclusion caused by postentrapment decrepitation resulted in H_2_O leakage.

Previous studies have shown that UHP inclusions could be affected by different degrees of postentrapment modification (7, 26–28), but other studies have pointed out that UHP inclusions with specific characteristics (e.g., negative crystals and high H_2_O volume fractions) are least affected by postentrapment modifications (29). Therefore, the degree of postentrapment modifications, especially fluid–host reaction, must be evaluated for the studied MFIs. To clarify the distribution of major elements between the inclusions and host minerals, we performed a mapping analysis of Si, Ca, and Fe. Major elements maps (Fig. 4) showed that the contact boundaries between MFIs and host minerals were very clear and straight and did not shown an obvious halo caused by a reaction or diffusion. Fluid leakage during the polishing process resulted in tiny gaps between the daughter and host minerals, which appeared as tiny low-content zones near contact boundaries (Fig. 4*B*) and were not caused by the postentrapment fluid–host reaction. Hence, the morphology (Fig. 3) and major element mappings (Fig. 4) of the MFI–host contact interfaces clearly demonstrate that fluid–host reactions and element migration were negligible. Meanwhile, the following observations are consistent with the features of inclusions minimally affected by postentrapment modifications (29): i) most inclusions are regular negative crystals with a moderate size of ~20 μm; ii) the regular morphology and relatively constant daughter mineral assemblages with similar phase proportion indicate crystallization from a single fluid rather than accidental entrapment or postentrapment modification; iii) the inclusion walls are flat and regular (Fig. 3) and do not present obvious signs of postentrapment re-equilibrium, as reported in a study by Ferrando et al. (7); and iv) most inclusions have high and constant water contents of ~40 wt.%, indicating negligible H_2_O diffusion and loss. Otherwise, the water content would be more variable. Although some inclusions in the omphacite and garnet have been subjected to different degrees of postentrapment burst and fluid leakage, burst and fluid leakage had no effect on the daughter mineral assemblages. The MFIs in garnet might have been somewhat more modified than those in omphacite, which was mainly because of H_2_O leakage rather than as fluid–garnet interactions (Fig. 4 *D*–*F*). Therefore, we assume that the MFIs experienced a low degree of postentrapment modification; thus, such modifications had a very limited impact on reconstructing the composition of the original captured fluid.

Theoretically, the mineral assemblages of MFIs in omphacite and garnet should be exactly the same; however, nonnegligible differences are observed between them. This significant difference cannot be the result of low-degree reaction. According to kinetics theory (30), crystal growth absorbs elements in solution, resulting in chemical heterogeneity between the fluid near the growing crystal surface and the fluid at more a distal location. Similarly, different host minerals absorb different elements. The distinct difference in the composition of locally captured fluid is related to the host mineral species. For example, diopside and grossular only exist in MFIs of omphacite and garnet, respectively. Therefore, crystallization and growth of different host minerals in the veining fluid lead to local heterogeneity of the fluid composition and differences in the daughter mineral assemblages between the MFIs in omphacite and garnet. In conclusion, differences in the daughter mineral assemblages between the MFIs in omphacite and in garnet are likely caused by the local chemical heterogeneity of the vein-forming fluids. Nevertheless, local heterogeneity of fluid occurs at the mineral scale, while the composition of the fluid is relatively homogeneous at the larger vein scale. Therefore, it is reasonable to conclude that the average composition obtained by the MFI analysis represents the early vein-forming fluid.

According to 3D modeling and calculations, the calculated average composition of the captured fluids can be estimated as 22.5 wt.% SiO_2_, 13.2 wt.% CaO, 7.0 wt.% SO_3_, 5.5 wt.% CO_2_, 3.4 wt.% Al_2_O_3_, 4.5 wt.% MgO, 1.3 wt.% TFe_2_O_3_, 0. 6 wt.% BaO, and 41.6 wt.% H_2_O, with trace amounts of Li, Na, and K. As shown in Table 1, the reconstructed fluid composition of MFIs is between a hydrous melt and an aqueous fluid (31), and it is consistent with the composition of supercritical fluid reported by Ni et al. (1), i.e., the concentrations of solute range from 30 to 70 wt.%. Manning’s experiment showed that the formation of supercritical fluid was closely related to the polymerization of silicate components in the fluid (32) and the degree of polymerization of silicate components in the fluid would increase with higher solute content (33). A series of related experiments showed that increases in temperature and pressure can significantly improve the solubility of silicate minerals in water (34–36). Under the pressure and temperature conditions of island arc (2.5 to 6.0 GPa, 700 to 900 °C), the fluid released by subducted slab can dissolve more than 20 to 30 wt.% of silicates (37), thus forming solute-rich aqueous fluid or supercritical fluid. The SiO_2_ content of the captured fluid in the MFIs clearly conforms to this range. For the critical H_2_O content parameter, our results are compatible with the supercritical fluid reported by Mibe et al. in their experiments at 3.6 GPa (H_2_O: 27 to 35 wt%) (3). These experimental data indicate that the trapped fluid by MFIs could be supercritical or close to supercritical. However, the high solubility of the fluid alone cannot be used to determine whether it is a supercritical fluid according to experimental petrology, e.g., the subsolidus aqueous fluid under the UHP condition can also dissolve a large number of insoluble elements (e.g., Si and Al) with similar composition characteristics as supercritical fluids (38). Compared with the recent experimental data on basalt–H_2_O solubility (38), the fluid composition (especially SiO_2_ and H_2_O) calculated by MFIs has values of 4 GPa and 900 °C to 5 GPa and 900 °C, thus indicating a subsolidus fluid composition, which is closer to that at 5 GPa and 900 °C. This means that the present study may have documented more of subsolidus aqueous fluids close to the SCEP.

For the solid phase-dominated metamorphic rock systems, the SCEP is the end point of wet solidus curves, and it controls the forming conditions of supercritical fluid (1). Whether peak temperatures and pressures experienced by the rock exceed the SCEP of the corresponding system is the most important index for judging whether supercritical fluids may exist. Previous studies have suggested that the pressure and temperature conditions for the formation of the studied veins may be ~3.2 GPa and ~800 °C, respectively (25). The strong experimental evidence suggested that the SCEP of the basalt–H_2_O system for producing supercritical fluid is at pressure >5.5 GPa and temperature >1,050 °C (5). The pressure and temperature conditions for the formation of the studied vein are lower than those of the SCEP of the basalt–water system. Although the results of Kessel (5) and Elazar (38) are very convincing, there are still some differences between the experimental system (basalt–H_2_O–CO_2_) and our natural samples (carbonate+sulfate+basalt–H_2_O). Therefore, there may be some discrepancies between the actual SCEP of the studied sample and the experimental results. Combined with Elazar’s experimental data (38), we determined that the inclusions should document subsolidus aqueous fluids close to the SCEP, which have extremely strong element migration ability similar to that of supercritical fluid. Although this is not a supercritical fluid in the strict sense of experimental petrology, it is quite close. Therefore, our sample can still provide significant reference guidance for an in-depth understanding of supercritical fluids in nature. In the present study, to distinguish “dilute” aqueous fluids formed under relatively low pressure and temperature conditions, UHP fluids with obvious supercritical fluid properties (strong element migration ability) formed under UHP conditions are referred to as generalized supercritical fluids. Of course, the generalized supercritical fluid may not strictly conform to the definition of “supercritical fluid” in terms of experimental petrology. The fluid documented by MFIs should belong to the generalized supercritical fluid as we defined. In this studied case, the presence of coesite in omphacite from the investigated vein also confirms that the vein formed under UHP conditions. Furthermore, the occurrence of rutile and garnet indicates relatively high concentrations of high-field-strength elements (HFSEs) and high-rare-earth elements in the vein-forming fluid, which is consistent with the high mobility of traditional immobile elements in supercritical fluid (3). Previous studies on the fractionation mechanism of Nb to Ta also suggested the occurrence of supercritical fluid in the Bixiling complex (20). In a summary of the above observation, we conclude that the MFIs in both the omphacite and garnet from Bixiling represent direct samples of supercritical fluid derived from deep subducting continental slab.

We noted that the absence of HFSEs enriched daughter minerals such as rutile in the investigated MFIs, which is inconsistent with the general enrichment of HFSEs in supercritical fluid (3). On the contrary, rutile in the vein is distributed along the vein wall in the form of large euhedral grains (Fig. 2). This is consistent with the quenching experiment observation that rutile crystallizes as the first phase from a supercritical fluid and subsequently grows along the capsule wall (39). We suggest that rutile is one of the earliest minerals that crystallized from primitive supercritical fluid and may even predate omphacite. Due to the very high distribution coefficient of HFSEs between rutile and supercritical fluid (up to several hundreds) (39), the crystallization of rutile will extract most of the HFSEs in the original supercritical fluid without changing its nature. Thus, although the MFIs may represent an early differentiated fluid rather than a primitive supercritical fluid, they still preserved the supercritical characteristic.

Our results indicate that the presence of MFIs (with high solutes and water) in UHP rocks can be an intuitive indicator for the preliminary identification of supercritical fluid activity. Other discoveries of multiphase inclusions with similar characteristics in UHP rocks from subduction zones worldwide (28) imply that the supercritical fluid in subduction zones may be quite common, which is also consistent with trace element indicators (e.g., Th/U and Nb/Ta) (20, 40).

Compared with the dilute slab-derived fluid (<15 wt.%) at forearc depths of <80 km (41), the supercritical fluid released by the slab is “more concentrated” and more efficiently dissolves and mobilizes materials in subducted slabs, with ~60 wt.% solute and extremely high densities (up to 1.6), as calculated by the present study. Therefore, supercritical fluid is an efficient transport medium for moving silicate-rich components from subducting slabs to the mantle wedge, and it affects the formation of arc magmatic rocks and the growth of continental crust (42).

In addition to the silicate components, large amounts of calcite (~10 wt.%) and anhydrite (15 to 20 wt.%) have been identified in the MFIs, indicating that the calculated supercritical fluid shows extreme high solubility for volatile elements such as carbon and sulfur. The solubility of carbon and sulfur in slab-derived fluids is pivotal to control and regulate the carbon and sulfur cycle in subduction zones. The solubility of calcite and anhydrite in the fluids is mainly controlled by pressureand differs by several orders of magnitude between the conditions under the surface and at UHP (34, 43). The solubility difference will directly affect the efficiency of carbon and sulfur cycling in subduction zones. For the carbon recycling of subduction zones, carbonate mineral dissolution occurs at almost every stage of subduction and has been documented from forearcs to subarcs (15, 41, 44). However, the contribution to the carbon cycle of fluids released at different depths is obviously different. As the pressure increases, slab-derived fluids evolve from aqueous fluid to supercritical fluid, whose carbon solubility can reach ~3 wt.% (5 to 6 GPa) (45), which is close to our results (~2 wt.%, Table 1). This may be due to silicate dissolution promoting carbon dissolution in subduction fluids (46). The recycling of carbon back to the surface depends on the carbon-carrying capacity of the fluid and melt in each subduction. Therefore, our data confirm that the supercritical fluid released during deep subduction can migrate the carbon hosted in slabs efficiently, thus improving the efficiency of carbon recycling in subduction zones. Prior studies also demonstrated that carbon from subducted slabs predominantly migrates under UHP conditions [e.g., MFIs from the Alpscontaining micro-diamonds (15)] rather than within relatively shallow depths (< 60 km) (47, 48). On the contrary, Li et al. (49) found that the main release of sulfur from subducted slabs takes place at 70 to 100 km and reaches a maximum at ca. 90 km (sulfur solubility as high as 1 wt.%). However, according to a recent model (49), sulfur released via aqueous fluid during slab dehydration is insufficient to explain the enrichment of sulfur in arc magma, which only accounts for 1/5 to 1/3 of the sulfur outflux through arc magma production (50). A new efficient sulfur transport mechanism is the key to solving this problem. Compared with previous model calculation results (46), our data from the MFIs show much higher sulfur mobility (~3 wt.%) in supercritical fluid than that in aqueous fluid. In addition, this fluid migrates reducing or oxidizing sulfur very well (50, 51). These results indicate that supercritical fluid can effectively compensate for the lack of sulfur migration via aqueous fluid released by slab dehydration. Although our data only represent continental crust-derived fluids, they still provide considerable reference significance for the global sulfur cycle. Based on the model from Li et al. (49), our rough estimate indicates that sulfur released by slab fluids may account for 15 to 20% of the input sulfur, which is higher than the previous estimate of ~6%. Furthermore, large quantities of carbon and sulfur could be transported into the overlying mantle by supercritical fluid, which is evident by inclusions containing carbon and sulfur-bearing daughter minerals in recrystallized zircons (40) and silicate minerals (e.g., orthopyroxene and garnet) (52, 53) in Dabieshan metasomatized mantle wedge peridotites. Eventually, the subducted carbon and sulfur return to the surface via magmatic activity and volcanic eruptions. In general, supercritical fluids may play an important role in every stage of the carbon and sulfur cycle in subduction zones.

The present study provides critical information for revealing the quantitative principal composition of supercritical fluid in subduction zones. Our data demonstrate the quantitative composition of supercritical fluids and solubility of carbon and sulfur. Moreover, the findings confirm the large-scale mobilization of carbon and sulfur by supercritical fluid released from slabs under UHP conditions. The extremely strong mobility of carbon and sulfur indicates that supercritical fluids play a vital role in the carbon and sulfur cycle. Moreover, the contribution of supercritical fluids to the deep carbon and sulfur cycle may have been significantly underestimated in the past.

## Methods

### EPMA Analysis.

The major element mapping analysis of MFIs and host minerals (omphacite, garnet) was undertaken at the CAS Key Laboratory of Crust-Mantle Materials and Environments at the University of Science and Technology of China, Hefei, using a JXA-8530F Plus Hyper Probe. The operating conditions for the mapping analyses were an accelerating voltage of 15 kV and a probe current of approximately 200 nA. For the element mapping, the spatial resolution and dwell time of single point acquisitions were 0.2 μm and 10 ms, respectively.

### Raman Analysis and 3D Scanning.

Laser Raman analysis of the MFIs in the omphacite and garnet was performed at the CAS Key Laboratory of Crust-Mantle Materials and Environments at the University of Science and Technology of China, Hefei, using a Nicolet FT-Raman 960 spectrometer with a 532-nm Ar laser excitation. 3D imaging of MFIs was performed using a WITec alpha300R confocal Raman microscope (WITec GmbH) with 532 nm cobalt laser excitation at the State Key Laboratory of Lithospheric Evolution, Institute of Geology and Geophysics, Chinese Academy of Sciences. The energy of the laser source was 25 mW, and the spectral collection range was 100 to 4,000 cm^−1^. For 3D scanning, the spatial resolution of single-point acquisitions along the *x* and *y* axis was 0.5 μm, while that along the *z* axis was 1 μm. The time of single-point acquisitions was 0.5 to 2 s, and the time of single-inclusion scanning was approximately 20 h.

### 3D Modeling and Reconstruction of the Fluid Composition.

The Raman data analysis was performed with WITec Project plus software. 3D imaging and modeling analyses of the MFIs were performed using Image J software. 3D modeling (for details, see *SI Appendix*, Fig. S5) can obtain the absolute volume of each daughter mineral phase in MFIs. Combined with the density of daughter minerals, the mass fraction of each mineral phase can be calculated. According to the ideal chemistry of daughter minerals, we can quantitatively calculate the composition characteristics of the fluid trapped during the crystallization of host minerals (for details, see *SI Appendix*, Table S1). The ideal chemistry of daughter minerals is as follows: calcite: CaCO_3_; quartz: SiO_2_; anhydrite: CaSO_4_; ferro-holmquistite: Li_2_(Fe^2+^_3_Al_2_)Si_8_O_22_(OH)_2_; barite: BaSO4; muscovite: KAl_2_(Si_3_Al)O_10_(OH)_2_; clinochlore: Mg_5_Al(AlSi_3_O_10_)(OH)_8_; pargasite: NaCa_2_(Mg_4_Al)(Si_6_Al_2_)O_22_(OH)_2_; diopside: CaMgSi_2_O_6_; actinite: Ca_2_(MgFe^2+^)_5_[Si_4_O_11_]_2_(OH)_2_; grossular: Ca_3_Al_2_(SiO_4_)_3_; and hematite: Fe_2_O_3_. The data source is https//rruff.info. We assumed that the fluid in the inclusions was pure water when calculating the composition of MFIs. Although it has been reported that the fluid inclusions of Bixiling contain high NaCl contents (21), the increase in fluid content is limited (about 5%), even assuming that the fluid of MFIs is a saturated NaCl solution. Nonetheless, the salinity of the fluid of MFIs was most likely lower than that of the saturated solution. Therefore, the difference between the actual composition and calculated fluid composition should be further narrowed. For the MFIs in garnet, considering the analysis time of Raman scanning, the set scanning area did not include the trailing and surrounding tiny fluid inclusions of the main inclusion. Due to the obvious fluid leakage and incompleteness of the scanning area of Inclusion 8 (Fig. 2*D*), the calculated water content must be lower than the true value. We assumed that the original water content was ~40 wt.% based on the constant water content of the MFIs. The water content of the trapped fluid (Inclusion 8) was corrected to 40 wt.% to reconstruct the composition of the fluid closer to the true value (for details, see *SI Appendix*, Table S1).

## Supplementary Material

Appendix 01 (PDF)

## Data Availability

All study data are included in the article and/or *SI Appendix*.
