# Supplementary material for: Supercritical fluid in deep subduction zones as revealed by multiphase fluid inclusions in an ultrahigh-pressure metamorphic vein
Source: Proc Natl Acad Sci U S A. 2023 May 8;120(20):e2219083120. doi: 10.1073/pnas.2219083120 (PMC10193987; doi:10.1073/pnas.2219083120)
Supplement: Supplementary file 1 — Appendix 01 (PDF) [file pnas.2219083120.sapp.pdf]

PNAS

Supporting Information for

Supercritical fluid in deep subduction zones as revealed by multiphase fluid inclusions in an ultrahigh-pressure metamorphic vein

Deshi Jin[1], Yilin Xiao[1,2], Dongbo Tan[1*], Xiaoxia Wang[1], Yangyang Wang[1], Wancai Li[1], Wen Su[3], Xiaoguang Li[3]*

*Corresponding author. **Email:** ylxiao@ustc.edu.cn; **Email:** dbtan@ustc.edu.cn

This PDF file includes:

Figures S1 to S6; Table S1

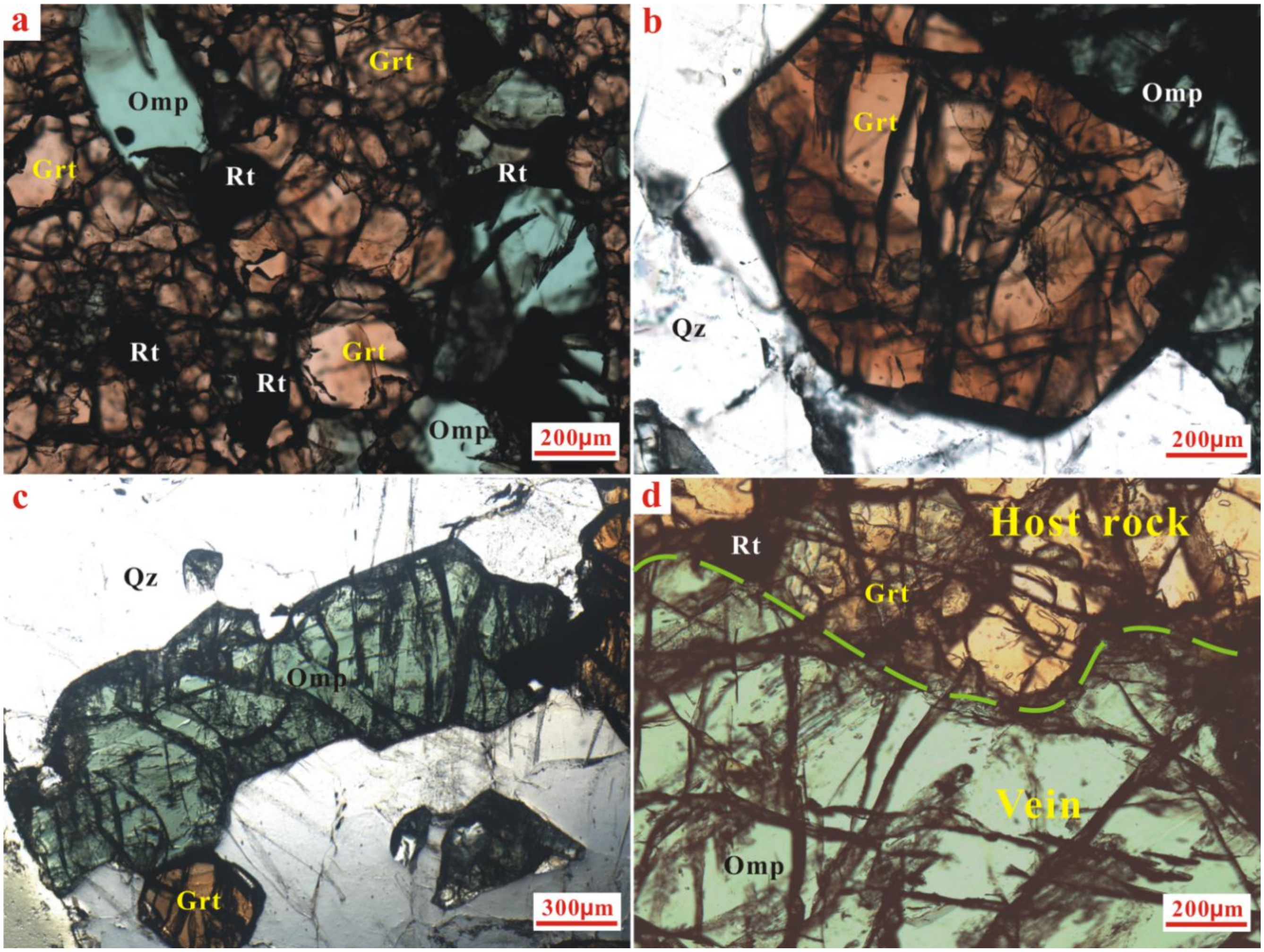

Fig. S1. Photographs of eclogites and the UHP vein in Bixiling. (a) Photograph of eclogites in Bixiling.The grain size of the minerals in the host eclogites is relatively small, and the crystal morphology is irregular. (b),(c) and (d) Photographs of garnet and omphacite in the UHP vein. The grain size and crystal morphology of the vein minerals are obviously better than those of the corresponding minerals in the surrounding eclogite.

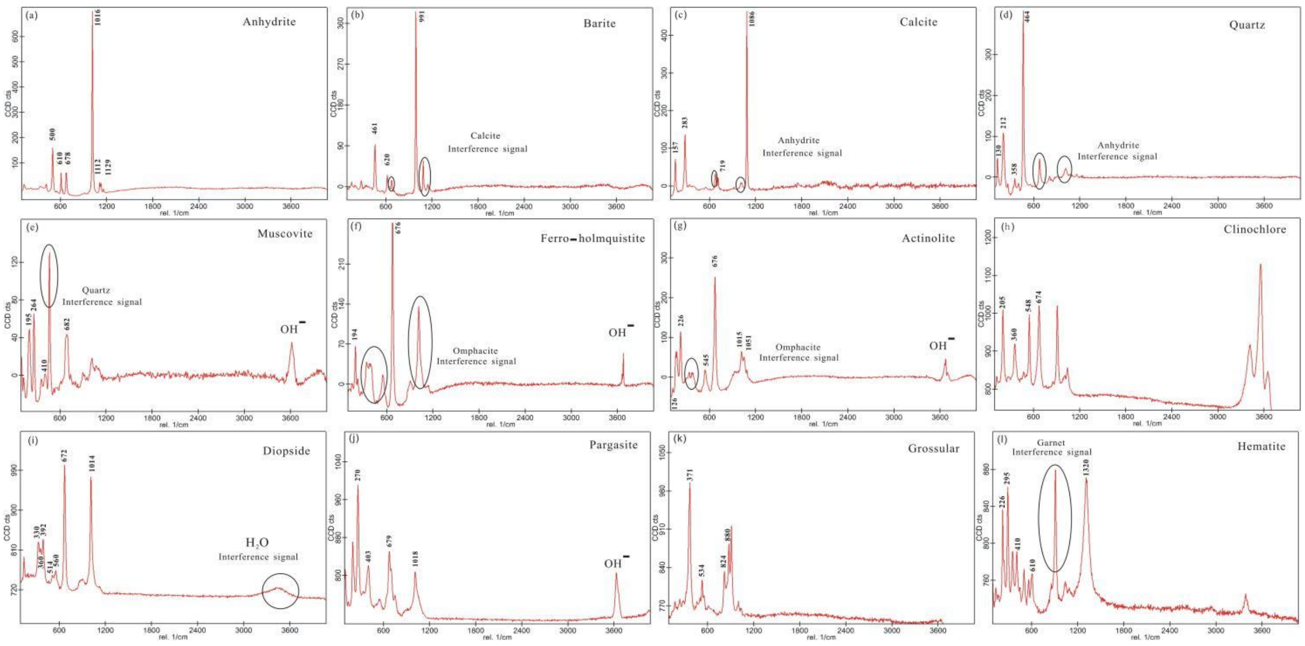

Fig. S2. Raman spectra of the daughter minerals within MFIs from the Bixiling UHP metamorphic vein.The peaks circled in the figures are interference signals of host minerals and other daughter minerals.

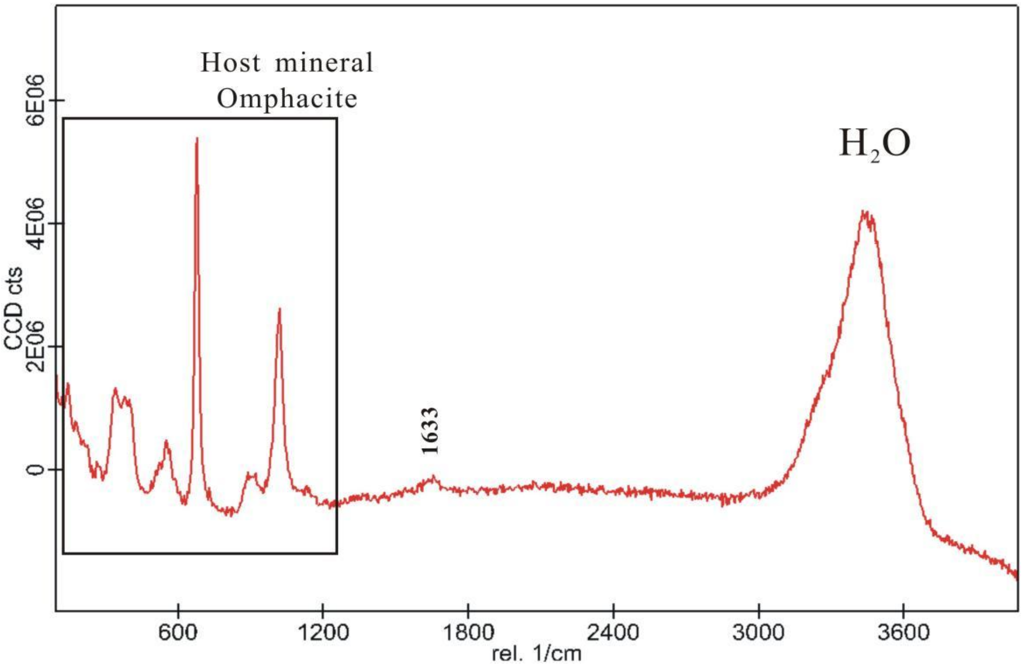

Fig. S3. Raman spectra of water within the MFIs from the Bixiling UHP metamorphic vein.

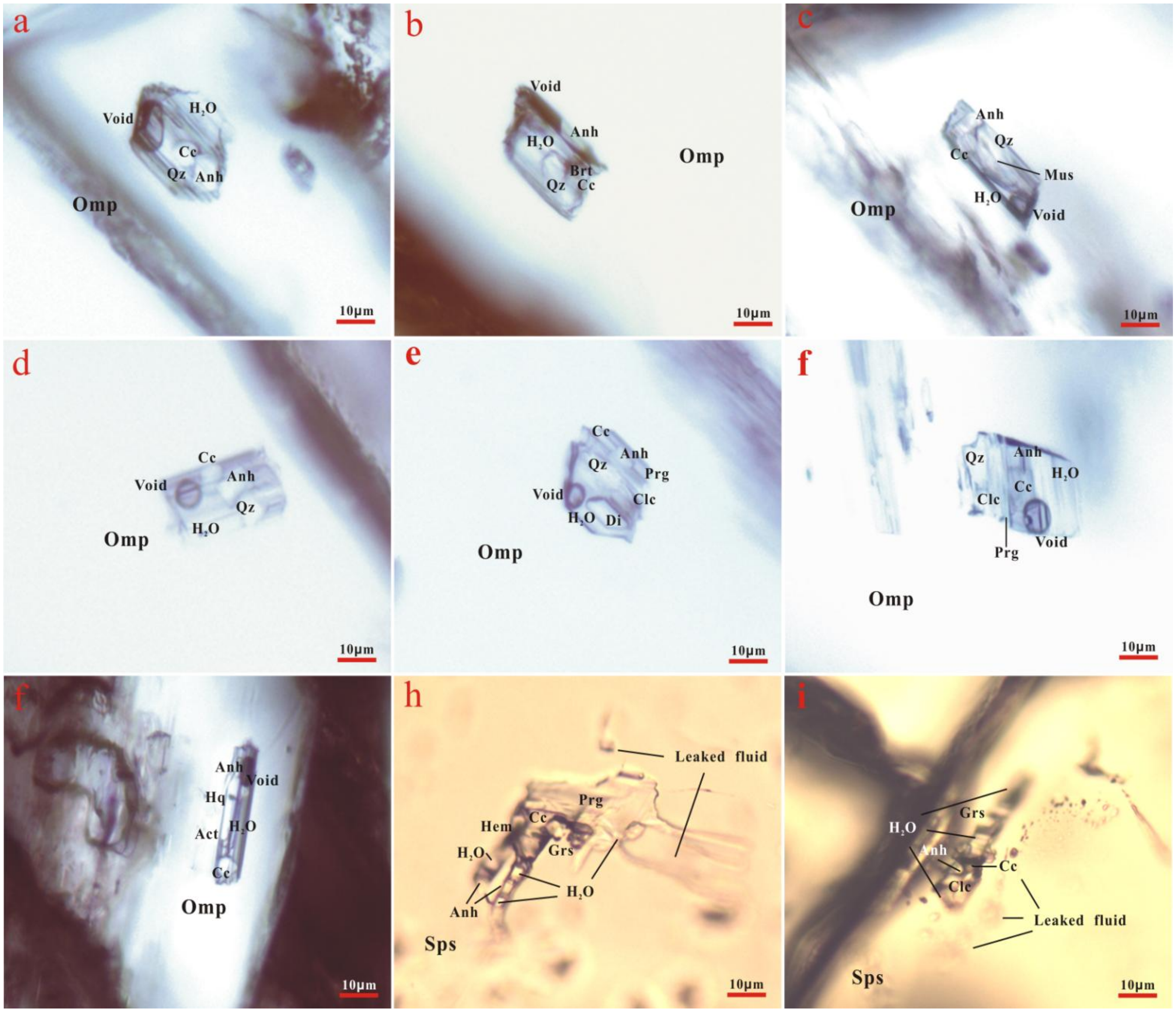

Fig. S4. Photomicrographs of the MFIs that have been 3D imaged and modeled within UHP complex metamorphic vein from Bixiling eclogite.

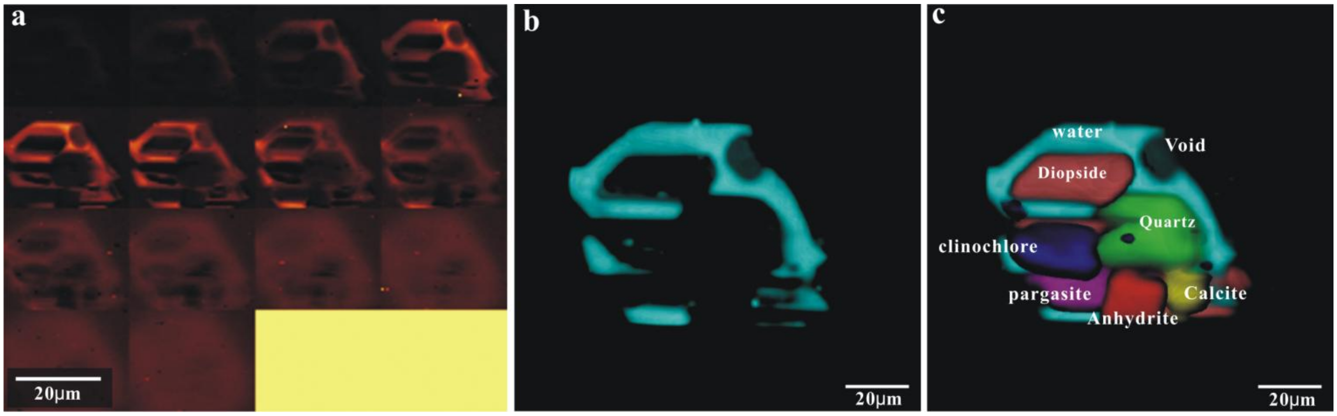

Fig. S5. Schematic diagram of 3D inclusion modeling. (a) Raman scanning shows the distribution of water in the inclusion from shallow to deep layers. (b) 3D modeling of water in the inclusion based on the results of scanning. (c) 3D distribution of mineral phases in the inclusion.

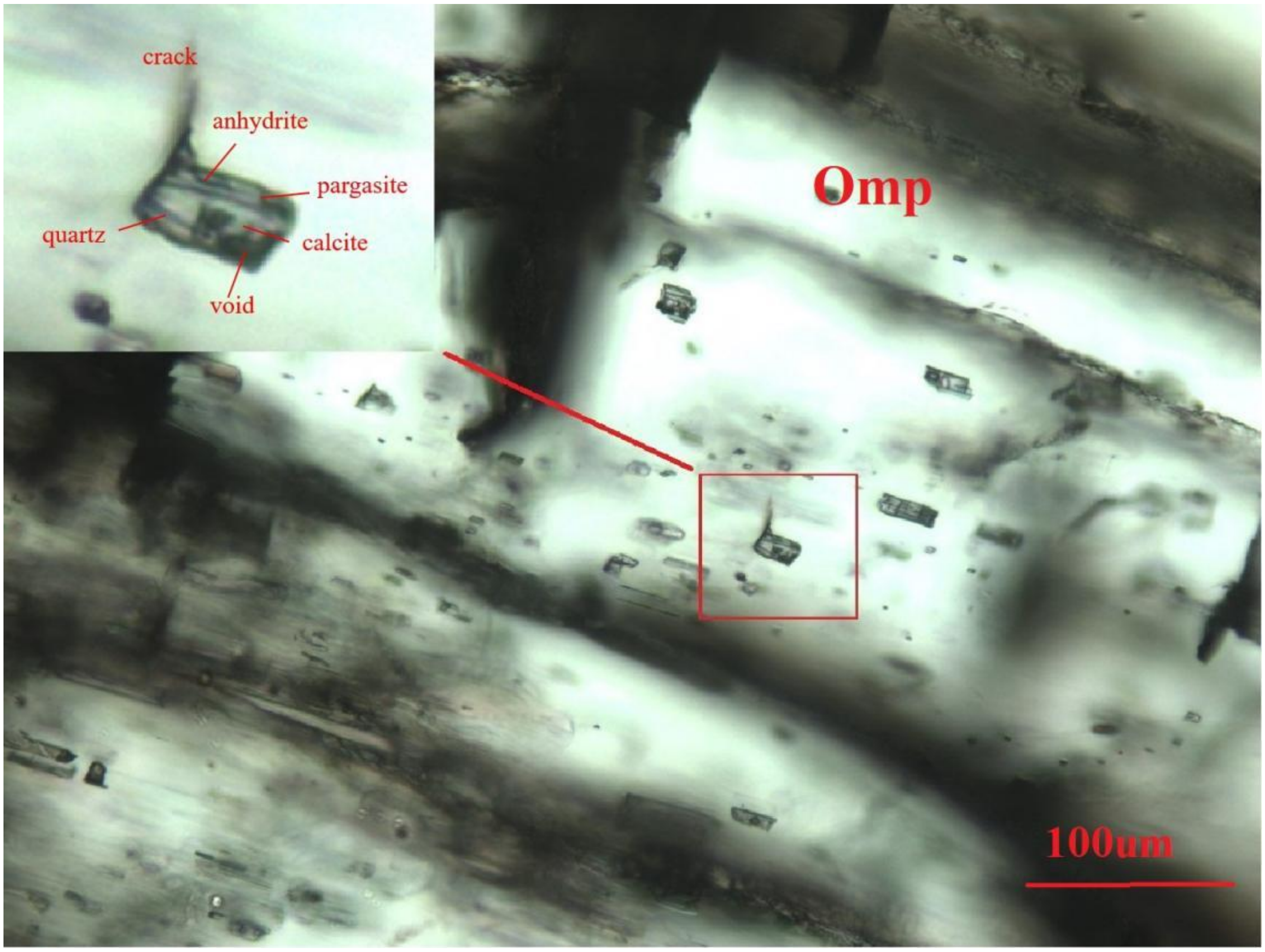

Fig. S6 Micrograph of the MFIs in omphacite. The MFIs show preferred directional distribution parallel to mineral elongation. The cracking inclusions present symbiosis with non-cracking inclusions in the same mineral grain. Raman analysis results show that such MFIs with leakage still have simar daughter minerals to MFIs with no leakage. Little water was detected in the cracked inclusions, leaving an irregular cavity.

Table S1. Summary table of calculation results of 3D modeling of MFIs

Host Mineral	Inclusion	Mineral Assemblages	Volume(μm^3)	Volume Fraction	Density(g/cm^3)	Mass(pg)	Mass Fraction(wt.%)	Density(g/cm^3)
Omphacite	1	calcite	1354	4.7%	2.71	3669.34	8.1%	1.57
		quartz	3698	12.9%	2.65	9799.70	21.7%	
		anhydrite	2633	9.2%	2.98	7846.34	17.4%	
		Ferro-holmquistite	1298	4.5%	3.10	4023.80	8.9%	
		water	19767	68.8%	1.00	19767.00	43.8%	
		total	28750	100.0%		45106.18	100.0%	
	2	calcite	1352	5.3%	2.71	3663.92	9.0%	1.60
		quartz	3765	14.8%	2.65	9977.25	24.5%	
		anhydrite	2018	7.9%	2.98	6013.64	14.8%	
		barite	824	3.2%	4.30	3543.20	8.7%	
		water	17566	68.8%	1.00	17566.00	43.1%	
		total	25525	100.0%		40764.01	100.0%	
	3	calcite	2247	7.7%	2.71	6089.37	12.4%	1.69
		quartz	5082	17.5%	2.65	13467.30	27.3%	
		anhydrite	2511	8.6%	2.98	7482.78	15.2%	
		muscovite	1527	5.3%	2.95	4504.65	9.1%	
		water	17710	60.9%	1.00	17710.00	36.0%	
		total	29077	100.0%		49254.10	100.0%	
	4	calcite	3587	8.0%	2.71	9720.77	13.6%	1.59
		quartz	5189	11.5%	2.65	13750.85	19.2%	
		anhydrite	5957	13.2%	2.98	17751.86	24.8%	
		water	30386	67.3%	1.00	30386.00	42.4%	
		total	45119	100.0%		71609.48	100.0%	
	5	calcite	1101	4.3%	2.71	2983.71	7.2%	1.61

		anhydrite	932	3.6%	2.98	2777.36	6.7%	
		quartz	2221	8.6%	2.65	5885.65	14.2%	
		clinochlore	883	3.4%	2.70	2384.10	5.7%	
		pargasite	1015	3.9%	3.20	3248.00	7.8%	
		diopside	2019	7.8%	3.30	6662.70	16.0%	
		water	17585	68.3%	1.00	17585.00	42.3%	
		total	25756	100.0%		41526.52	100.0%	
	6	calcite	3519	8.6%	2.71	9536.49	13.8%	1.69
		anhydrite	1082	2.7%	2.98	3224.36	4.7%	
		quartz	4300	10.5%	2.65	11395.00	16.5%	
		clinochlore	4377	10.7%	2.70	11817.90	17.1%	
		pargasite	2549	6.3%	3.20	8156.80	11.8%	
		water	24950	61.2%	1.00	24950.00	36.1%	
		total	40777	100.0%		69080.55	100.0%	
	7	calcite	981	10.4%	2.71	2658.51	18.9%	1.49
		anhydrite	377	4.0%	2.98	1123.46	8.0%	
		actinolite	653	6.9%	3.10	2024.30	14.4%	
		Ferro-holmquistite	409	4.3%	3.10	1267.90	9.0%	
		water	7000	74.3%	1.00	7000.00	49.7%	
		total	9420	100.0%		14074.17	100.0%	
Garnet	8	calcite	1200	20.4%	2.71	3252.00	21.8%	2.54
		anhydrite	700	11.9%	2.98	2086.00	14.0%	
		grossular	212	3.6%	3.82	809.84	5.4%	
		pargasite	1910	32.5%	3.20	6112.00	41.0%	
		hematite	200	3.4%	5.00	1000.00	6.7%	
		water	1653	28.1%	1.00	1653.00	11.1%	
		total	5875	100.0%		14912.84	100.0%	
	8*	calcite	1200	9.2%	2.71	3252.00	14.7%	1.69
		anhydrite	700	5.4%	2.98	2086.00	9.4%	

	grossular	212	1.6%	3.82	809.84	3.7%	
	pargasite	1910	14.6%	3.20	6112.00	27.7%	
	hematite	200	1.5%	5.00	1000.00	4.5%	
	water	8840	67.7%	1.00	8840.00	40.0%	
	total	13062	100.0%		22099.74	100.0%	
9	calcite	200	7.2%	2.71	542.00	10.8%	1.81
	anhydrite	29	1.1%	2.98	86.42	1.7%	
	grossular	71	2.6%	3.82	271.22	5.4%	
	clinochlore	970	35.1%	2.7	2619.00	52.3%	
	water	1490	54.0%	1	1490.00	29.7%	
	total	2760	100.0%		5008.64	100.0%	
